# Microglia targeting by adeno-associated viral vectors

**DOI:** 10.3389/fimmu.2024.1425892

**Published:** 2024-07-05

**Authors:** Maria Stamataki, Björn Rissiek, Tim Magnus, Jakob Körbelin

**Affiliations:** ^1^ ENDomics Lab, Department of Oncology, Hematology & Bone Marrow Transplantation, University Medical Center Hamburg-Eppendorf, Hamburg, Germany; ^2^ Department of Neurology, University Medical Center Hamburg-Eppendorf, Hamburg, Germany

**Keywords:** microglia, adeno-associated virus (AAV), purinergic signaling, gene targeting, neuroinflammation

## Abstract

Microglia play a crucial role in maintaining homeostasis of the central nervous system and they are actively involved in shaping the brain’s inflammatory response to stress. Among the multitude of involved molecules, purinergic receptors and enzymes are of special importance due to their ability to regulate microglia activation. By investigating the mechanisms underlying microglial responses and dysregulation, researchers can develop more precise interventions to modulate microglial behavior and alleviate neuroinflammatory processes. Studying gene function selectively in microglia, however, remains technically challenging. This review article provides an overview of adeno-associated virus (AAV)-based microglia targeting approaches, discussing potential prospects for refining these approaches to improve both specificity and effectiveness and encouraging future investigations aimed at connecting the potential of AAV-mediated microglial targeting for therapeutic benefit in neurological disorders.

## 1 Introduction

As resident immune cells of the central nervous system (CNS), microglia play a pivotal role in CNS homeostasis. Microglia most likely derive from progenitor cells in the yolk sac during early embryonic development and migrate into the CNS during fetal development. Once located in the brain or spinal cord, they proliferate and differentiate into mature microglia (1). While the earlier accepted dichotomy of proinflammatory M1 and anti-inflammatory M2 microglia is now being questioned as microglia phenotypes change throughout time at a rapid pace and acquire the demanded phenotype depending on their surroundings (2), their activation is being triggered via diverse signaling molecules, including neurotransmitters, cytokines, and purines (3). Purines and purinergic signaling play a pivotal role in the regulation of microglial function, making it a crucial pathway to understand in the context of neuroinflammation. Microglia express various purinergic receptors, such as P2X (4–6) and P2Y (7) receptors, which respond to the release of ATP and other purines from damaged or stressed cells. Activation of these receptors can trigger microglial activation, migration, phagocytosis, and the release of pro-inflammatory cytokines, which are essential for the clearance of pathogens and damaged cells (8). However, dysregulation of purinergic signaling in microglia has been involved in the progression of neurological conditions such as neuroinflammation (4), ischemia (9, 10) or Alzheimer’s disease (11). The genetic targeting of these cells, therefore, will grant us a better understanding of their role in the disruptions of CNS homeostasis. Although the selective genetic targeting of microglia remains a technical challenge, recent advances in viral vector development have paved the way for cell-type specific gene transfer.

## 2 Genetic targeting using the adeno-associated virus

The adeno-associated virus (AAV) has emerged as one of the most promising gene delivery platforms in preclinical research (12). AAV-mediated gene expression is a multi-step process requiring AAV attachment to the cell surface, internalization, lysosomal transport and escape, nuclear entry, release of the viral genome (uncoating), and successful replication of the ssDNA genome that finally enables the transcription into coding RNA (13). In principle, each of the mentioned steps can be targeted to enhance AAV-mediated microglial gene expression. While modifications of the viral capsid can influence its affinity for the cell surface, its internalization into the cell as well as its intracellular processing (14), the use of microglia-specific promoter and enhancer elements can ensure targeted gene transcription (15, 16). The specificity of gene expression can be further increased on a post-transcriptional level by using target sequences for cell-specific miRNAs (16, 17).

**Figure 1:**
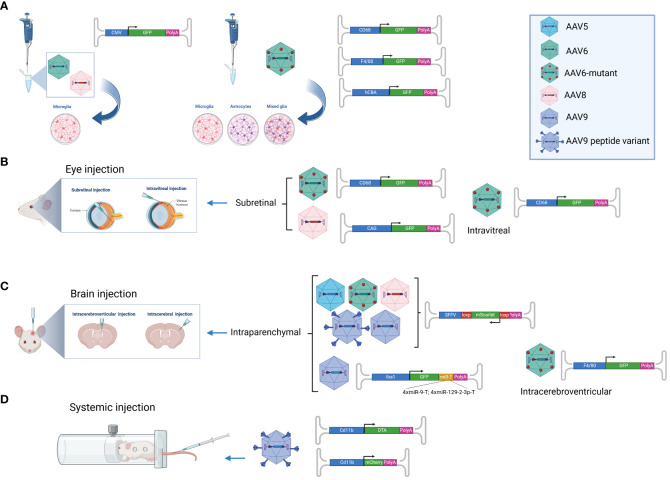
AAV-based microglia targeting approaches. Overview of AAV vectors that have successfully been used to transduce microglia. **(A)** AAV6 and AAV8 were shown to efficiently transduce primary microglia *in vitro* when employing the ubiquitous CMV promoter (18), while the AAV6 capsid mutant AAV TM6 in combination with the F4/80 or CD68 promoter showed specificity for microglia upon incubation with primary microglia, astrocytes and mixed neuroglia (19). **(B)** The AAV6 capsid mutants AAV TM6 and AAV6Δ4 showed successful microglia transduction *in vivo* upon intravitreal and subretinal injection when employing the CD68 promoter (21). AAV8 in combination with the ubiquitous CAG promoter was shown to transduce microglia upon intravitreal injection (20). **(C)** AAV5, AAV TM6, AAV8, AAV9 and the AAV9 peptide-displaying variants MG1.1 and MG1.2 successfully transduced microglia upon intraparenchymal brain injection while assessing transgene (mScarlet) expression by inducible vectors in cre-positive mice (24). AAV9-mediated microglia-specific transgene expression upon intraparenchymal brain injection was further enhanced by employing the Iba1 promoter and targeting elements for miR-9 and miR-129-23 in four copies each (16). **(D)** The AAV9 peptide-displaying variants ALAVPFR, ALAVPFK, HGTAASH, and YAFGGEG were recently shown to transduce microglia and macrophages with high efficiently even upon intravenous injection in mice. Gene expression was accomplished by employing the CD11b promoter (25). *This figure was generated with Biorender.com*.

So far, microglia targeting with AAV vectors has either been studied *in vitro* using primary rodent cells (15, 18, 19) or *in vivo* using rodent models. Multiple *in vivo* studies have investigated retinal microglia (20–23) probably because local AAV administration (subretinal or intravitreal) restricts the viral biodistribution to the eye, thereby preventing off-target transduction (e.g. of hepatocytes) and increasing the contact time between AAV particles and microglia which might enhance transduction efficiency. For similar reasons, most of the *in vivo* studies on brain microglia have been conducted upon local administration, intraparenchymal (16, 24) or intracerebroventricular (19). Until very recently, to our knowledge, no studies were reporting on microglia-targeted AAVs upon systemic administration *in vivo*. This changed last year with a groundbreaking study by Young et al. (25) that will certainly facilitate future research on microglia. A graphical overview of different AAV targeting approaches is provided in **Figure 1**.

### 2.1 Transduction of microglia by natural AAV serotypes

Several natural AAV serotypes have been assessed for their potential to transduce microglia *in vitro*. Su et al. investigated the transduction efficiency of AAV2, 5, 6, 8 & 9 in neonatal and adult microglia cultures from C57/BL6 mice, employing the strong and ubiquitous CMV promoter (18). Microglial transgene expression was achieved with all tested AAV serotypes. While the expression strengths of AAV2, AAV5 and AAV9 ranged at similar level, AAV6 and AA8 yielded more profound transgene expression. Upon incubation with primary cells, AAV6 showed an 80-fold higher and AAV8 a 25-fold higher microglial transgene expression compared to AAV2 (18). Conflicting results, however, were reported in another study, claiming that primary neonatal mouse microglial were not permissive for AAV1-9 as well as for AAVRh10, whereas mixed neuroglia cultures showed neuronal and astrocytic transgene expression upon incubation with the same vector preparations (19). The reason for the observed differences remains unknown but might be related to potential differences in the used vector amount or other non-reported experimental details. The ability of AAV8 to transduce microglia, at least, was recently also confirmed *in vivo*. Chandler et al. showed that, subretinal injection of recombinant AAV8 vectors does not only induce ocular infiltration of peripheral immune cells but also mediated AAV-derived GFP expression in retinal microglia by use of the strong and ubiquitous CAG promoter (20). While all of the above-mentioned studies utilized non-specific promoters, cell type-specific promoter elements are a possible way to limit transgene expression to microglia. Certain elements in the 5’ untranslated regions of the genes for Hexosaminidase B and CD86 have been shown to allow for a strong and more selective gene expression after transfecting the human and murine microglia cell lines C20 and SIM-A9 (15).

In another approach AAV9 vectors were equipped with a 1.7-kb promoter region derived from the myeloid cell-specific gene Iba1, as well as two sets of 4-tandem copies complementary to miR-9 and miR-129-2-3p (16). Vectors were applied by direct parenchymal brain injection. Analysis conducted one-week post-injection indicated successful microglial transduction by the Iba1 promoter-driven vector constructs which was accompanied by substantial transduction of non-microglia cells especially in the cerebral cortex. While only about 2% of GFP-positive cells in the cerebral cortex expressed the monocyte marker Iba1, about 70% and 85% of GFP-positive were co-stained for Iba1 in the striatum and cerebellum, respectively. Addition of the targeting elements for miR-9 and miR-129-2-3p drastically increased targeting specificity resulting in 85% targeting specificity for the cerebral cortex as well as 100% specificity for striatum and cerebellum. This was confirmed by immunolabeling of the brain sections for Iba1, transgenic GFP as well as TMEM119, a highly specific microglial marker not expressed by CNS macrophages, dendritic cells, infiltrating monocytes, or other immune or neural cell types. Moreover, possible transduction of astrocytes was excluded by immunostaining for GFAP, proving absence of GFAP/GFP double-positive cells (16).

### 2.2 Point mutations in the AAV capsid that enhance microglia transduction

In 2014 Pandya et al. developed an AAV6 triple mutant T492V/Y705F/Y731F (=TM6) to optimize intracellular trafficking, thereby enhancing transduction of monocyte-derived dendritic cells (26). The group of Chakrabarty compared the triple mutant AAV TM6 to the serotypes AAV1-9 as well as AAVRh10 on primary mouse mixed neuroglial and microglia cultures (19). Indeed, AAV6 TM6 in combination with the promoters F4/80 or CD68 enabled efficient microglial transduction and gene expression *in vitro*. Upon intracerebroventricular injection in mice, microglia transduction was most specific using the F4/80 promoter (about 70-80% of GFP-positive cells were also positive for Iba1) but overall transduction efficacy was modest (19). Another group, investigated the same TM6 vector construct with CD68 promoter *in vivo* within the retinal environment (21). In their *in vivo* experiments, Maes et al. utilized adult C57BL6/J mice and a mouse model of photoreceptor degeneration (Pde6b^rd10/rd10^). Different delivery routes, including subretinal and intravitreal injections, were used to introduce the AAV to adult mice to target microglia in distinct retinal layers. Further, the effect of optic nerve crush on microglial transduction by AAV was investigated. While optic nerve crush failed to enhance microglial transduction, the delivery route strongly influenced layer-specific transduction of microglia. Intravitreal AAV injection was shown to favor microglial transduction in the inner retinal layers, whereas subretinal injection was favored microglial transduction in the outer plexiform layer (OPL). Flow cytometry revealed mean transduction efficiencies of 45% and 25% for intravitreal and subretinal injections, respectively. In a next step, Maes et al. introduction four additional mutations (K459S, K493S, K531E, R576Q) in the AAV capsid that had been shown to reduce AAV binding to heparan sulfate proteoglycan (HSPG). The resulting AAV6^Δ4^ variant demonstrated a two-fold increase in OPL microglia transduction in certain analyzed regions, whereas microglia transduction in the inner plexiform layer remained unchanged indicating limitations in lateral diffusion (21).

### 2.3 Directed evolution of AAV capsid libraries

Inserting targeting peptides into the receptor binding region of the AAV capsid is an efficient way to re-direct viral tropism to novel cellular target structures and the screening of so-called random AAV display peptide libraries has already yielded multiple tissue- or cell type-specific AAV variants (27–31). In a study conducted in 2022 Lin et al. report on the directed evolution of AAV vectors for efficient gene delivery to microglia (24). They applied a Cre-based AAV targeted evolution strategy (32) and employed a peptide library displayed on the capsid of AAV9 between the amino acids A588 and 589 that was screened over two selection rounds on primary murine microglia. The two most strongly enriched AAV variants, AAV-cMG.QRP and AAV-cMG.WPP, were tested, showing higher efficiency than the wild type but still only labelling a small proportion of microglia (24). To improve transduction, a peptide library of additional AAV-cMG.WPP variants was generated through semi-random mutagenesis and screened *in vivo* upon intraparenchymal brain injection into Cx3cr1^CreER^ mice. Two highly enriched capsid variants, named AAV-MG1.1 and AAV-MG1.2, were identified after two subsequent screening rounds. These variants demonstrated strong and widespread mScarlet expression in all brain areas tested when packaged with a Cre-dependent reporter vector and injected into the brains of Cre-positive mice (24). The transduction efficiency of AAV-MG1.1 and AAV-MG1.2 was higher than that of parental AAV-cMG.WPP capsid as well as of AAV5, AAV8, AAV9 and the already discussed AAV TM6 variant harboring the Y731F/Y705F/T492V triple mutation (19). Furthermore, the transgene expression driven by AAV-MGs was restricted to microglia, as confirmed by selective labelling in different mouse models. While these AAV-MGs showed enhanced *in vivo* transduction of microglia, they did not effectively transduce cultured mouse microglia.

A similar approach, also employing an AAV9-displayed peptide library, was conducted very recently (25). In the study by Young et al. (25), however, the AAV display peptide library was screened *in vivo*, upon systemic injection in mice. The systemic screening approach allowed for the identification of AAV variants that, for the first time, enable blood-brain barrier penetration followed by subsequent transduction of CNS microglia. Several of the identified novel AAV variants demonstrate remarkable efficiency in transducing microglia *in vivo*. The two most efficient AAV variants, displaying the peptides YAFGGEG and HGTAASH, achieved transduction rates of up to 80% which is higher than what has ever been achieved before. Unfortunately, though, these variants lack specificity. Despite using the phagocyte-specific Cd11b promoter, Young et al. observed more than 5% positive neurons and oligodendrocytes, 12.5% positive astrocytes as well as over 30% positive endothelial cells. As microglia only represent a small fraction of all brain cells, the absolute number of transduced neurons, oligodendrocytes and astrocytes in fact will be far higher than the number of transduced microglia. Further, the AAV variants identified by Young et al. do not distinguish between CNS microglia and tissue-resident macrophages, all of which are transduced quite efficiently. Potential off-target transduction of other cell types in peripheral organs has not been evaluated. Nevertheless, this breakthrough for the first enables global modulation of microglial gene expression *in vivo* and therefore holds great potential for biomedical research.

## 3 Perspectives

The studies reviewed here have collectively demonstrated significant progress in the development of microglia-targeted AAV vectors. These developments hold great promise for potential therapeutic applications in neurological disorders. The utilization of novel AAV variants capable of efficiently transducing these cell populations *in vivo* represents a significant step forward in the field of gene therapy. However, despite these notable advancements, several limitations of current studies should be acknowledged and addressed.

One limitation is the requirement for local administration of many AAV vectors (16, 19–24, 33). While the so-far described locally administered microglia-targeted AAVs only achieve moderate transduction efficacy, systemic injection requires high target specificity to avoid adverse effects such as liver toxicity and thrombocytopenia. Although systemic injection has been investigated by Young et al., the desired specificity in distinguishing between microglia and non-microglial cells has not been achieved yet (25). To overcome this limitation, future screening efforts of AAV libraries could employ more stringent cell sorting techniques. For example, instead of solely isolating single cells with anti-CD11b antibody (monocytes), screening could be repeated with a focus on sorting for CD45 low/intermediate cells that also express high levels of CD11b (CD45^low^, CD11b^high^) (34). The introduction of additional point mutations at positions such as W503 within the AAV9 capsid (35) might help to further limit off-target transduction. Such refined approach may enhance the specificity of AAV vectors for microglial targeting while minimizing off-target effects. Since AAV tropism can also be re-targeted by the insertion of specific nanobodies into the AAV capsid (36), nanobodies against cell surface molecules highly expressed by microglia such as the P2X7 receptor (6) might serve as additional AAV targeting moieties in the future.

It is also important to note that all studies conducted thus far have been performed in rodent models. While rodents serve as valuable models for basic and preclinical research, there is a need to validate the efficacy and safety of current AAV variants in non-human primates (NHPs) or human tissue samples. Inter-species differences in AAV tropism may exist, requiring further investigation (29, 37–39). Alternatively, screening efforts could be expanded to include AAV capsid libraries in NHPs or human tissue to identify novel variants with enhanced specificity and efficacy for microglial targeting.

In conclusion, while recent studies have made significant advances in developing AAV vectors for microglia, important challenges and opportunities for improvement remain. Addressing the limitations will be essential for advancing the field and appreciating the full therapeutic potential of microglial-targeted gene therapy.

## Author contributions

MS: Visualization, Investigation, Writing – review & editing, Writing – original draft. BR: Visualization, Writing – review & editing, Writing – original draft. TM: Funding acquisition, Writing – review & editing, Writing – original draft, Conceptualization. JK: Writing – review & editing, Writing – original draft, Supervision, Funding acquisition, Conceptualization.
